# Multimodal fusion of EHR and ECG based on deep learning for predicting new-onset coronary heart disease in cancer patients

**DOI:** 10.3389/fcvm.2026.1834641

**Published:** 2026-06-17

**Authors:** Sheng Zhang, Wei Wang

**Affiliations:** 1 Key Laboratory of Digital-Intelligent Disease Surveillance and Health Governance, North Sichuan Medical College, Nanchong, Sichuan, China; 2 Department of Cardiology, Chongqing University Cancer Hospital, Chongqing, China

**Keywords:** cancer, coronary heart disease, deep learning, electrocardiogram, multimodal fusion

## Abstract

**Background:**

Cancer patients carry elevated risk of new-onset coronary heart disease (CHD), but accurate risk stratification remains limited. We aimed to develop a multimodal deep learning model for predicting new-onset CHD in cancer patients.

**Methods:**

Consecutive cancer patients from January 2010 to December 2020 were enrolled. The primary endpoint was new-onset CHD during hospitalization or follow-up. We constructed a hybrid CNN-LSTM model by fusing electronic health records (EHRs) and 12-lead electrocardiogram (ECG). Model performance was compared with seven machine learning methods (LR, DT, KNN, SVM, RF, AdaBoost, GBDT). Clinical utility and calibration were assessed by ROC, decision curve analysis (DCA) and calibration curves. Subgroup analyses were conducted in early-stage (Stage II) and late-stage (Stage III + IV) cohorts.

**Results:**

A total of 1262 patients were included in the final analysis, of whom 722 (57.2%) developed new-onset CHD, with 696 (55.2%) classified as early-stage (Stage II). The CNN-LSTM model achieved an AUC of 0.975 (95%CI: 0.962–0.988), outperforming seven conventional machine learning models. In subgroup analysis, the model yielded an AUC of 0.924 in early-stage patients and 0.888 in late-stage patients. SHAP analysis identified that PAB, CRP, age, cTnI, PT, and ECG markers including HR, QT and QTc intervals were the strongest predictive features.

**Conclusion:**

The multimodal CNN-LSTM model enables robust and interpretable prediction of new-onset CHD in cancer patients. Coagulation, myocardial injury, inflammatory, metabolic, and electrophysiological markers provide mechanistic insights and may facilitate early cardioprotective strategies.

## 1 Introduction

Cancer patients are at a significantly higher risk of developing coronary heart disease (CHD) due to both cancer-related treatments and underlying cardiovascular risk factors (1, 2). The morbidity and mortality associated with cardiovascular diseases are notably elevated in cancer patients, with studies showing that up to 30% of cancer patients with CHD die—a mortality rate up to ten times higher than the general population (3). Consequently, early identification of cancer-associated CHD (Ca-CHD) is critical for improving the prognosis and survival rates of these patients.

Clinical assessment of CHD typically involves circulating biomarkers, electrocardiography (ECG), and echocardiography (UCG). Biomarkers such as cardiac troponin (cTn), natriuretic peptides (NPs), and C-reactive protein (CRP) provide substantial evidence for assessing cardiovascular risk (4–6). cTn and NPs, in particular, are considered among the most promising markers for cardiovascular risk stratification in cancer patients (7). For instance, elevated cTn levels following anthracycline therapy correlate with adverse outcomes and cardiac dysfunction (8), while increased NPs are associated with heart failure and reduced ejection fraction (9). A high-sensitivity CRP shows a negative predictive value of 94.1% for trastuzumab-induced cardiotoxicity (10). However, a comprehensive risk prediction tool specific for Ca-CHD has not yet been established. Recent studies have shown the superiority of multi-modal prediction approaches. A multicenter study combining cTnI, NT-proBNP, ST2, and echocardiographic parameters, such as LVEF and myocardial strain, improved cardiovascular risk prediction (11). Furthermore, abnormalities like QTc prolongation and arrhythmias are commonly observed in Ca-CHD patients (12), additional risk factors include sex, age, and resting heart rate (13, 14), highlighting the need for a comprehensive assessment integrating various biomarkers and clinical features.

The growing volume of electronic health records (EHRs) and the rise of deep learning (DL) present promising avenues for the comprehensive early detection of Ca-CHD. Several studies have successfully integrated clinical data and AI techniques for predicting cardiovascular risks (15). For instance, Cai et al. developed a combined machine learning framework to identify drug-induced cardiovascular complications, achieving AUCs between 0.784 and 0.842 (16). Similarly, a deep learning-based dual-screening tool applied to 2,085 lung cancer patients reached an AUC of 0.871 for predicting cardiovascular mortality (17). DL models like convolutional neural network (CNN), long short-term memory (LSTM) models were used to predict heart disease risk in cancer patients based on longitudinal EHRs data outperformed baseline models in predicting congestive heart failure, coronary artery disease, cardiomyopathy, myocardial infarction, transient ischemic attack, and aortic regurgitation, with AUC scores ranging from 0.719 to 0.955 (17–20).

Notably, AI models utilizing multi-lead ECG signals have shown strong performance. For example, Liu et al. proposed a multi-lead convolutional neural network (ML-CNN) for myocardial infarction detection, achieving a sensitivity of 95.4%, specificity of 97.37%, and accuracy of 96% (21). Similarly, Bairmani et al. proposed a framework (CNN-Bi-LSTM-transformer) achieved high diagnostic performance, robustness to class imbalance, and human-level interpretability simultaneously, providing a reliable, scalable solution for automated ECG analysis (22). Researches have further verified the outstanding classification performance of CNN in ECG-based cardiac disease identification, with binary classification accuracy up to 98.35%–99.3%. Such performance demonstrates the superior feature extraction and recognition efficiency of CNN-based frameworks (23). The low hardware cost and real-time inference capability endow CNN-enabled ECG analysis systems with great application potential in long-term and continuous cardiac monitoring scenarios (24–26).

These studies underscore the potential of AI-driven models with multi-modal data to enhance cardiac risk stratification in cancer patients. However, further refinement of algorithms is needed to better integrate heterogeneous risk factors for Ca-CHD. In response, we propose a hybrid CNN-LSTM model that combines clinical notes (text), laboratory results (numerical data) from EHRs, and 12-lead ECG signals for more accurate identification of Ca-CHD.

## 2 Methods

### 2.1 Study procedure

This was a retrospective observational cohort study conducted at a single cancer center. All patients diagnosed with malignant tumor who received chemotherapy, targeted therapy, immunotherapy, or radiotherapy during the study period were initially screened. Patients were enrolled if they had no documented coronary heart disease (CHD) at admission and had complete baseline clinical, laboratory, and electrocardiographic data. Those with severe cardiovascular disease, missing key variables, or incomplete follow-up records were excluded. The endpoint was defined as new-onset CHD during hospitalization or follow-up, diagnosed in accordance with current clinical guidelines based on typical manifestations, electrocardiographic alterations, and cardiac biomarkers. Electrocardiographic parameters included heart rate, PR interval, QRS duration, QTc interval, ST-segment changes, and T-wave abnormalities, and the last available ECG during hospitalization was used for assessment.

### 2.2 Data preprocessing

**Figure 1:**
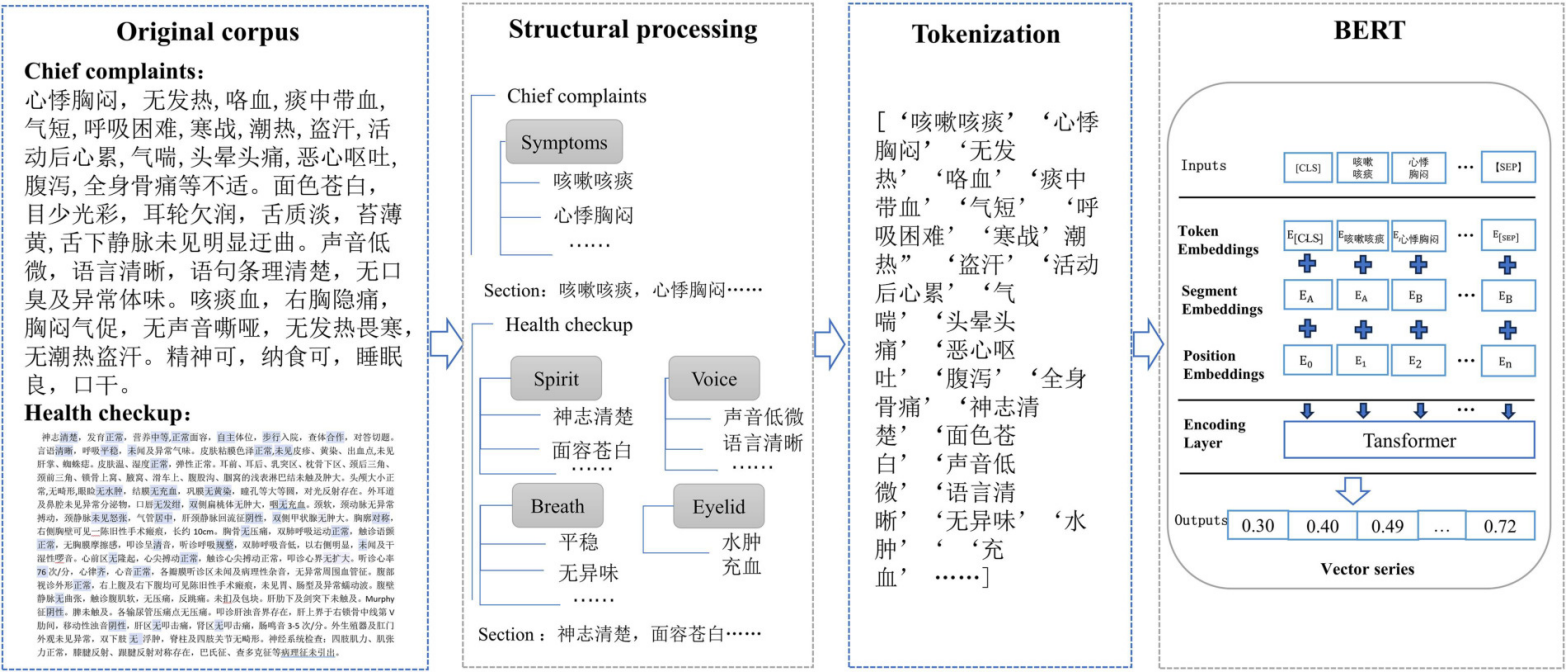
Diagram showing a four-step natural language processing workflow for medical text in Chinese: original corpus extraction, structural processing into symptom and health checkup categories, tokenization into word units, and encoding with BERT transformer to produce output vector series.

The Bidirectional Encoder Representation from Transformers (BERT) model (27) is a popular tool for extracting semantic information of the text in EHRs since it has a stronger capability of representing the semantic information of text. We obtained the vector representation of the clinical corpus by using the BERT encapsulated library Bert-as-Service, provided by Tencent AI Lab (28). The raw text corpus preprocessing description using BERT was illustrated in Figure 1.

The clinical indicators were extracted from the patient's basic information, personal history, physical examination, laboratory examination, cardiac ultrasound report, diagnostic results. They were processed in a structured way including data cleaning, conversion and integration to address cases of missing, inconsistent and outlier values. Missing data with a missing rate < 50% were managed using multiple imputation by chained equations (MICE), and variables with a missing rate ≥ 50% were excluded from analysis. Assignment method was used to encode the counting data as (0, 1) in numerical form.

For ECG signals, we used the Daubechies D6 (db6) wavelet basis function to eliminate the noise and remove baseline drift. The detail components of D1 (250–500 Hz), D2 (125–250 Hz), D3 (62.5–125 Hz) and the approximation component of A10 (0–0.4875 Hz) were removed. The remaining components were reconstructed to obtain a noise-free signal. The Pan-Tompkins algorithm (29) was then used to detect the R-peaks and the QRS-wave detection was used to segment the denoised signal. Each segmented heartbeat consists of 600 sampling points, with 200 points from the left side of the R peak and 399 points on the right side. Each heartbeat covered the range of a P-QRS-T wave for 0.6 seconds. Finally, the 12-lead heart beats were normalized to prepare the inputs for the network.

### 2.3 Model framework

**Figure 2:**
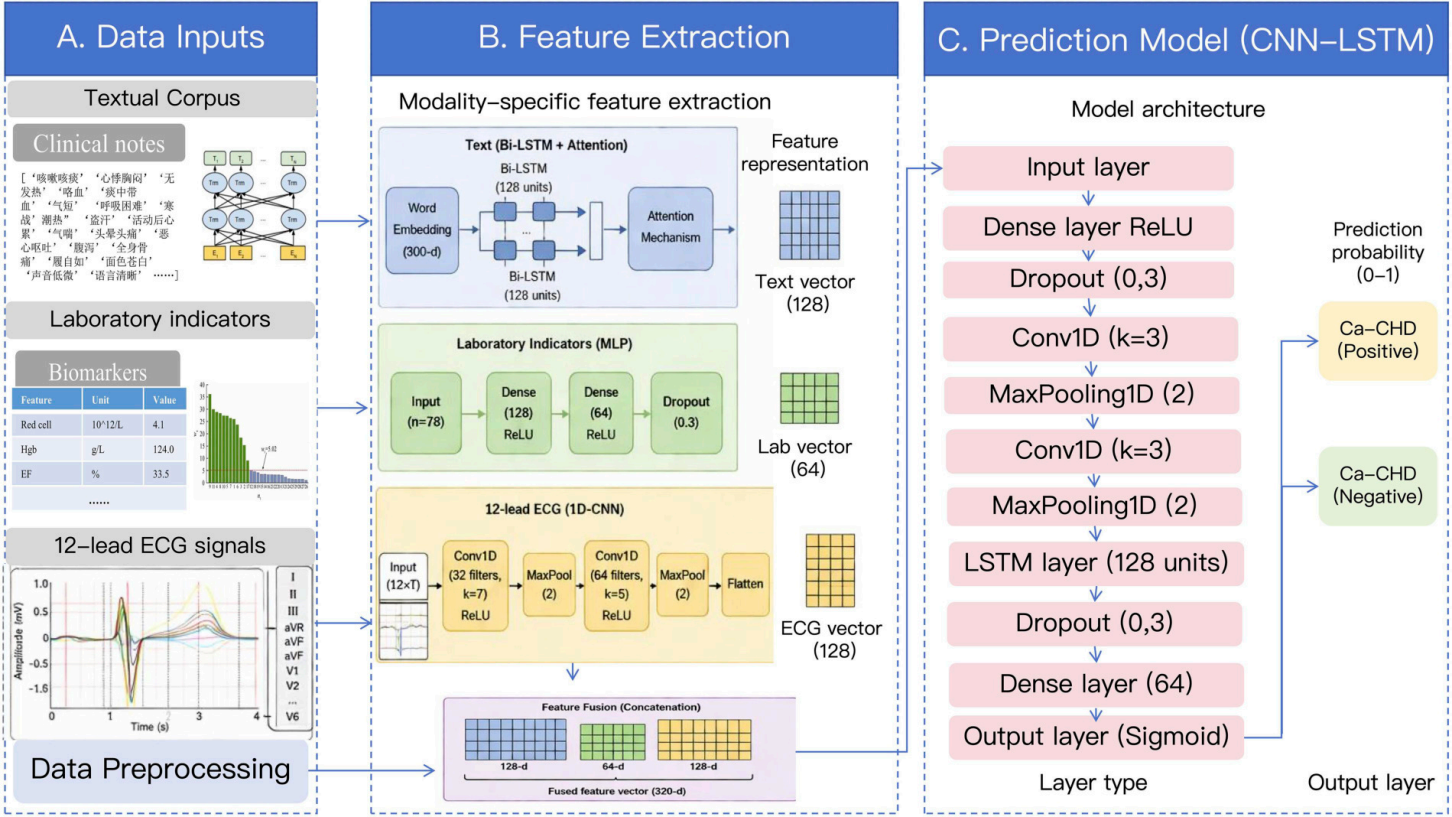
Flowchart illustrating a machine learning pipeline for coronary artery–coronary heart disease (Ca-CHD) prediction, showing three sections: A. Data Inputs (clinical notes, laboratory biomarkers, 12-lead ECG signals); B. Feature Extraction (text vectors via Bi-LSTM, lab vectors via dense layers, ECG vectors via 1D-CNN, then concatenation); C. Prediction Model (neural network architecture with dense, convolutional, max pooling, LSTM, and sigmoid output layer for Ca-CHD positivity or negativity).

A multimodal CNN–LSTM model was developed to predict cancer-associated new-onset CHD (Ca-CHD). As shown in Figure 2, the model integrates three types of preprocessed inputs: textual clinical notes, structured laboratory indicators, and 12-lead ECG signals. In the feature extraction stage, textual data are first embedded and processed using a Bi-LSTM with an attention mechanism to produce a 128-dimensional text vector. Laboratory indicators are passed through a multi-layer perceptron (MLP) consisting of two dense layers (128 and 64 units) with dropout, generating a 64-dimensional lab vector. ECG signals are processed using a 1D-CNN with sequential convolution and max-pooling layers, followed by flattening to yield a 128-dimensional ECG vector.

These modality-specific vectors are concatenated into a 320-dimensional fused feature vector, which is then sequentially processed by fully connected dense layers and an LSTM layer (128 units) to capture both spatial and temporal dependencies. A final sigmoid output layer generates the probability of Ca-CHD, classifying patients as cancer with new-onset CHD or cancer without new-onset CHD. Model performance was evaluated using accuracy, F1-score, and the area under the receiver operating characteristic (ROC) curve (AUC) with 95% confidence intervals (CIs). Calibration curves, Brier score, and decision curve analysis (DCA) were applied to assess calibration and clinical utility. Subgroup analyses were performed stratified by cancer stage.

The convolutional layer first perceives each feature locally and then performs a comprehensive operation on the local part at a higher level to obtain global information. The input data are convolved with a set of kernels of different shapes to generate discriminant feature maps for recognition representation. An activation function is introduced to generate the nonlinear decision boundary through a nonlinear combination of weighted inputs (30). All data embeddings are stacked in a region matrix $M\in R^{d\times |V|}$ where |V| is the size of a region, and *d* is the dimensionality of data. In each region, we used *L* convolutional filters to learn the local features. A filter $F_{l}(1\leq l\leq L)$ generates the feature map $\;y_{n}^{l}\;$ as (Equation 1) follows,

$$y_{n}^{l}=f(W^{l}x_{n:n+\omega -1}+b^{l})$$ (1)

Where $W\in R^{d\times |V|}$ and *b* denote the convolution kernel and network bias vectors respectively, ω is the length of the filter, f is the ReLU function. When a filter gradually traverses from $x_{1:\omega -1}$ to $x_{n+\omega -1:n}$, we obtain the output feature maps $y^{l}=y_{1}^{l},y_{2}^{l},\cdots ,y_{n-\omega +1}^{l}$ of filter $F_{l}$.

Long-short term memory (LSTM) network is one of the most effective gated recurrent neural networks (RNN) for practical applications. The basic unit of LSTM is represented by cells. The input, forgetting, and output gates control the behavior of the cells to achieve the long-term storage of memory information (31). The weight of the self-loop is controlled by the forgetting gate $f_{i}^{\left(t\right)}$ (time *t*, cell i) (Equation 2) and the Sigmoid cell sets the weight to a value between 0 and 1,

$$\;\;\;\;\;\;\;\;\;\;\;\;\;\;\;f_{i}^{\left(t\right)}=\sigma \left(b_{i}^{f}+\sum_{j}U_{i,j}^{f}x_{j}^{\left(t\right)}+\sum_{j}W_{i,j}^{f}h_{j}^{\left(t-1\right)}\right)$$ (2)

Where $\;x^{\left(t\right)}$ is the current input vector, $h^{t}\;$ is the current hidden layer vector, $\;h^{t}$ contains the output of all LSTM cells. b,U,W are the cycle weights of the offset, the input weight and the forgetting gate respectively. External input gate unit $S_{i}^{\left(t\right)}$ (Equation 3) is updated in a manner similar to the forgetting gate as mentioned above,

$$\;\;\;\;\;\;\;\;\;\;\;\;\;\;\;\;\;\;\;S_{i}^{\left(t\right)}=\sigma \left(b_{i}^{s}+\sum_{j}U_{i,j}^{s}x_{j}^{\left(t\right)}+\sum_{j}W_{i,j}^{s}h_{j}^{\left(t-1\right)}\right)$$ (3)

The output $h_{i}^{\left(t\right)}$ of LSTM cells can also be closed by the output gate $q_{i}^{\left(t\right)}$ (Equation 4),

$$h_{i}^{\left(t\right)}=\tanh(S_{i}^{\left(t\right)})q_{i}^{\left(t\right)},$$

$$\;\;\;\;\;\;\;\;\;\;\;\;\;\;\;\;\;\;\;q_{i}^{\left(t\right)}=\sigma \left(b_{i}^{o}+\sum_{j}U_{i,j}^{o}x_{j}^{\left(t\right)}+\sum_{j}W_{i,j}^{o}h_{j}^{\left(t-1\right)}\right)$$ (4)

## 3 Results

### 3.1 Study population

**Figure 3:**
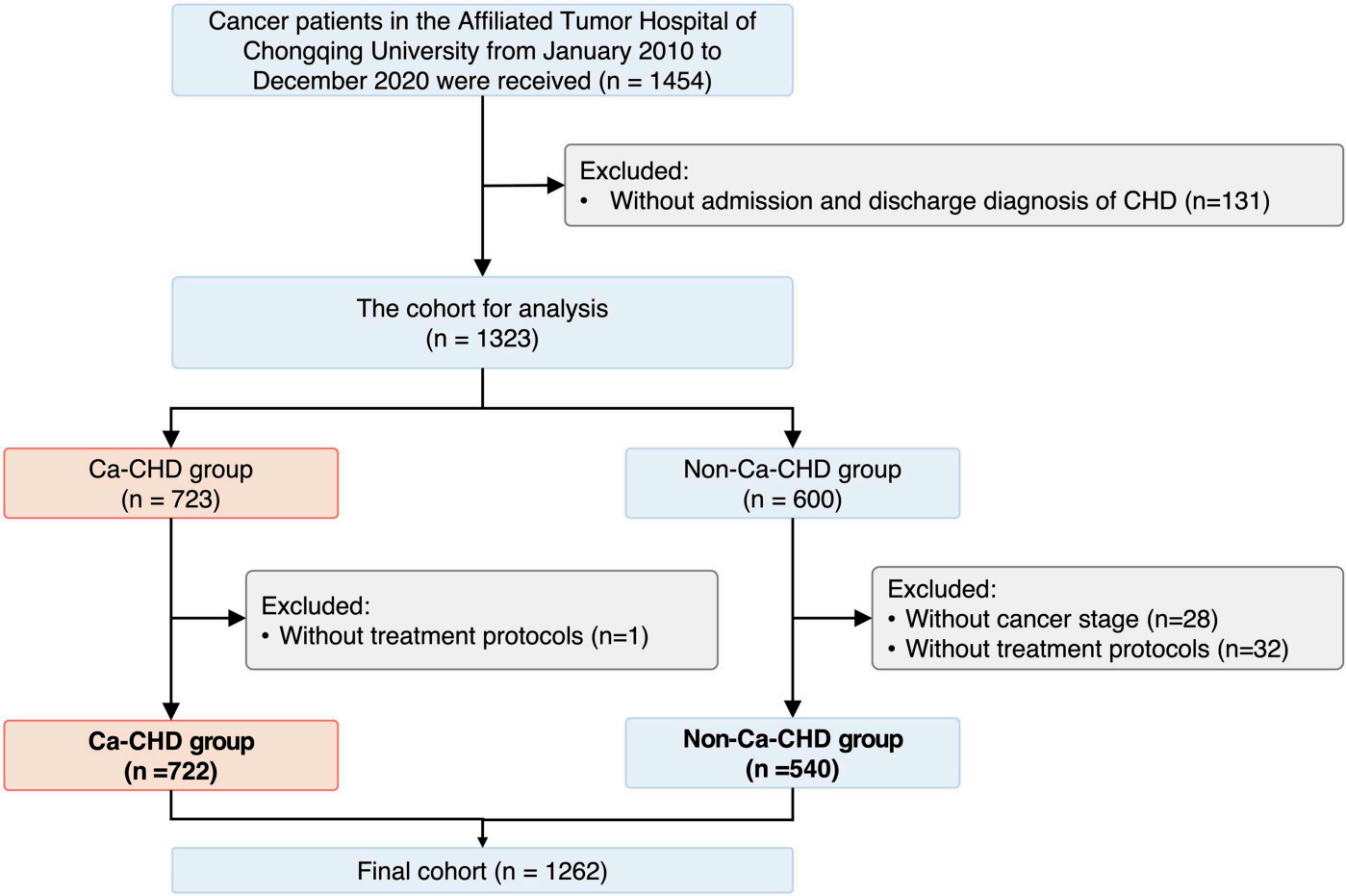
Flowchart illustrating patient selection in a retrospective cohort study. Out of 1,454 cancer patients, 131 were excluded. Of 1,323 analyzed, 723 were Ca-CHD and 600 Non-Ca-CHD; further exclusions led to a final cohort of 1,262 patients.

A total of 1454 patients in the Affiliated Tumor Hospital of Chongqing University from January 2010 to December 2020 who were diagnosed as cancer in admission clinical notes were collected, of which 131 without admission and discharge diagnosis of CHD. The remaining 1,323 eligible patients were stratified into two groups: 723 patients with new-onset CHD (Ca-CHD) and 600 patients without new-onset CHD (Non-Ca-CHD), of whom 1 case in Ca-CHD (without treatment protocols), 28 cases in Non-Ca-CHD (without cancer stage) and 32 cases (without treatment protocols) were excluded. Therefore, the final 1262 datasets for analysis in this study constituted with 722 Ca-CHD and 540 Non-Ca-CHD (Figure 3).

### 3.2 Procedure characteristics and outcomes

The baseline characteristics of patients, which were partially extracted from the 105 structured clinical features, were compared between the cancer with Ca-CHD group and the Non-Ca-CHD group. The Mann–Whitney U test was used for continuous data, and the chi-square test or Fisher's exact test was used for categorical variables. Categorical variables were presented as frequencies (percentages), and continuous variables were expressed as the median (interquartile range, IQR).

As presented in Table 1, significant between-group differences were observed in several baseline demographic and clinical characteristics between patients with cancer-associated new-onset coronary heart disease (Ca-CHD) and those without CHD. Patients in the Ca-CHD group were significantly older than those in the non-CHD group (median age: 73.00 [66.00–77.00] vs. 71.00 [62.00–77.00], *P* = 0.002). However, no significant differences were observed in sex distribution (*P* = 0.351), Karnofsky Performance Status (KPS) score (*P* = 0.275), or body mass index (BMI) (*P* = 0.526) between the two groups.

**Table 1:** Baseline demographic and clinical characteristics.

Characteristics	Ca-CHD (*n* = 722)	Non-Ca-CHD (*n* = 540)	*P*-value
Age, years	73.00 (66.00–77.00)	71.00 (62.00–77.00)	0.002
Sex, male, %	392 (54.3%)	278 (51.5%)	0.351
KPS score	82.49 (77.75–90.00)	81.06 (77.41–90.00)	0.275
BMI, Kg/m2	23.12 (21.67–24.73)	23.11 (21.48–24.64)	0.526
Cancer Type			
Lung cancer	216 (29.9%)	228 (42.2%)	<0.001
Prostate cancer	83 (11.5%)	112 (20.7%)	<0.001
Ovarian cancer	67 (9.3%)	65 (12.0%)	0.120
Colorectal cancer	59 (8.2%)	25 (4.6%)	<0.001
Gastric cancer	36 (5.0%)	14 (2.6%)	<0.001
Cancer stage, *n* (%)			
Ⅱ	373 (51.7%)	323 (59.8%)	0.004
Ⅲ	21 (2.9%)	6 (1.1%)	0.025
Ⅳ	328 (45.4%)	211 (39.1%)	0.018
Other complications, *n* (%)			
Hypertension	306 (42.4%)	247 (45.7%)	0.257
Diabetes	193 (26.7%)	188 (34.8%)	0.002
Hyperlipemia	38 (5.3%)	25 (4.6%)	0.703
Hypercoagulability	29 (4.0%)	33 (6.1%)	0.116
Medication			
Aspirin, clopidogrel, statin	317 (43.9%)	189 (35.0%)	<0.001
Paclitaxel, carboplatin	115 (15.9%)	119 (22.0%)	<0.001
Pemetrexed, cisplatin	150 (20.8%)	121 (22.4%)	<0.001
Pembrolizumab	44 (6.1%)	64 (11.9%)	<0.001
Cephalosporin, quinolone	96 (13.3%)	47 (8.7%)	<0.001

Regarding cancer types, significant differences were identified. The proportion of lung cancer and prostate cancer was significantly higher in the non-CHD group (both *P* < 0.001), whereas colorectal cancer and gastric cancer were more prevalent in the Ca-CHD group (both *P* < 0.001). No statistically significant difference was observed for ovarian cancer (*P* = 0.120). In terms of cancer stage, patients with Ca-CHD were more likely to present with advanced disease. Specifically, stage III and IV cancers were more frequent in the Ca-CHD group, while stage II disease was more common in the non-CHD group (all *P* < 0.05). For comorbidities, diabetes was significantly more prevalent in the non-CHD group (*P* = 0.002), whereas no significant differences were found for hypertension, hyperlipemia, or hypercoagulability. Regarding medication use, several treatment regimens differed significantly between the two groups. Patients in the Ca-CHD group had a higher proportion of cardiovascular-related medications (aspirin, clopidogrel, statins) (*P* < 0.001), as well as antibiotic use (cephalosporin, quinolone) (*P* < 0.001). In contrast, chemotherapy and immunotherapy regimens such as paclitaxel plus carboplatin and pembrolizumab were more frequently used in the non-CHD group (all *P* < 0.001).

As shown in Table 2, differences in baseline laboratory characteristics were observed between the Ca-CHD and non-Ca-CHD groups. Compared with the non-Ca-CHD group, patients in the Ca-CHD group exhibited significantly higher levels of low-density lipoprotein cholesterol (LDL), total bile acid (TBIL), cardiac troponin I (cTnI), prothrombin time (PT), fibrin degradation products (FDP), and a lower stroke volume (SV) (all *P* < 0.05). In particular, coagulation-related markers, including elevated PT and FDP, together with increased D-dimer levels (though not statistically significant), suggest a tendency toward coagulation and fibrinolytic system activation in the Ca-CHD group. Additionally, the higher LDL and cTnI levels reflect more pronounced dyslipidemia and myocardial injury in patients with Ca-CHD. These findings indicate that Ca-CHD patients may present with a more adverse cardiometabolic and prothrombotic profile compared with non-Ca-CHD.

**Table 2:** Baseline laboratory characteristics.

Characteristics	Ca-CHD (*n* = 722)	Non-Ca-CHD (*n* = 540)	*P*-value
Hematological parameters			
RBC, 10^12/L	4.18 (3.78–4.49)	4.14 (3.74–4.49)	0.210
HGB, Hemoglobin, g/L	125.46 (113.28–135.93)	125.90 (112.50–136.00)	0.784
MCV, fl	91.60 (88.82–94.33)	91.68 (88.95–94.50)	0.702
WBC,10^9/L	6.18 (5.04–7.40)	6.34 (5.11–7.73)	0.162
PLT,10^9/L	200.17 (162.40–239.84)	201.23 (162.15–244.43)	0.807
Lipid profile			
TC, mmol/L	4.48 (4.39–4.92)	4.48 (4.36–4.77)	0.567
TG, mmol/L	1.58 (1.23–1.58)	1.58 (1.24–1.58)	0.655
LDL, mmol/L	3.29 (2.53–3.43)	3.12 (2.34–3.43)	0.003
HDL, mmol/L	1.68 (1.34–1.81)	1.58 (1.27–1.81)	0.004
Liver function			
ALT, U/L	18.11 (13.20–24.70)	18.47 (12.80–24.78)	0.922
AST, g/L	22.18 (18.10–27.42)	21.90 (17.15–28.98)	0.311
GGT, U/L	32.05 (19.38–59.29)	30.50 (18.89–54.92)	0.233
TBIL, Total bile acid, umol/L	11.10 (8.19–14.38)	10.61 (7.68–13.88)	0.009
Renal function			
Creatinine, umol/L	63.17 (54.00–73.97)	62.59 (52.58–72.54)	0.194
CysC, umol/L	1.23 (1.01–1.35)	1.19 (0.97–1.39)	0.329
UA, umol/L	304.24 (267.66–356.68)	302.18 (262.45–353.55)	0.285
Cardiac biomarkers			
CKMB, U/L	14.69 (14.00–14.69)	14.69 (14.38–14.69)	0.579
cTnI, ng/L	1.88 (1.43–1.88)	1.88 (0.83–1.88)	< 0.001
BNP, pg/mL	76.23 (76.23–76.23)	76.23 (76.23–76.23)	0.615
Coagulation markers			
D-dimer, mg/L	0.85 (0.33–3.42)	0.75 (0.33–2.52)	0.569
PT, sec	11.27 (10.71–12.90)	10.28 (10.70–11.49)	0.002
APTT, sec	27.51 (25.70–29.08)	27.47 (25.80–29.08)	0.891
FDP, mg/L	3.58 (2.50–5.08)	2.92 (2.33–5.08)	0.008
Cardiac function			
EF, %	68.53 (67.26–69.70)	68.53 (67.00–69.54)	0.205
SV, mL/beat	69.44 (62.00–70.12)	69.44 (55.93–69.53)	0.004
FS, %	43.61 (38.06–52.32)	43.42 (37.99–52.32)	0.967
CO, L/min	5.81 (4.87–5.89)	5.70 (4.68–5.89)	0.356

### 3.3 Model settings

The preprocessed dataset for each patient, comprising textual embeddings, laboratory indicators, and 12-lead ECG signals, was randomly divided into training, validation, and testing sets at a ratio of 3:1:1. The CNN–LSTM model was trained using the Adam optimizer with a learning rate of 1 × 10⁻^3^, a batch size of 8, and for 50 epochs. All experiments were implemented in Python (version 3.14) using TensorFlow on a workstation with an Intel Core i5 processor and 32 GB RAM.

### 3.4 Model comparison

**Figure 4:**
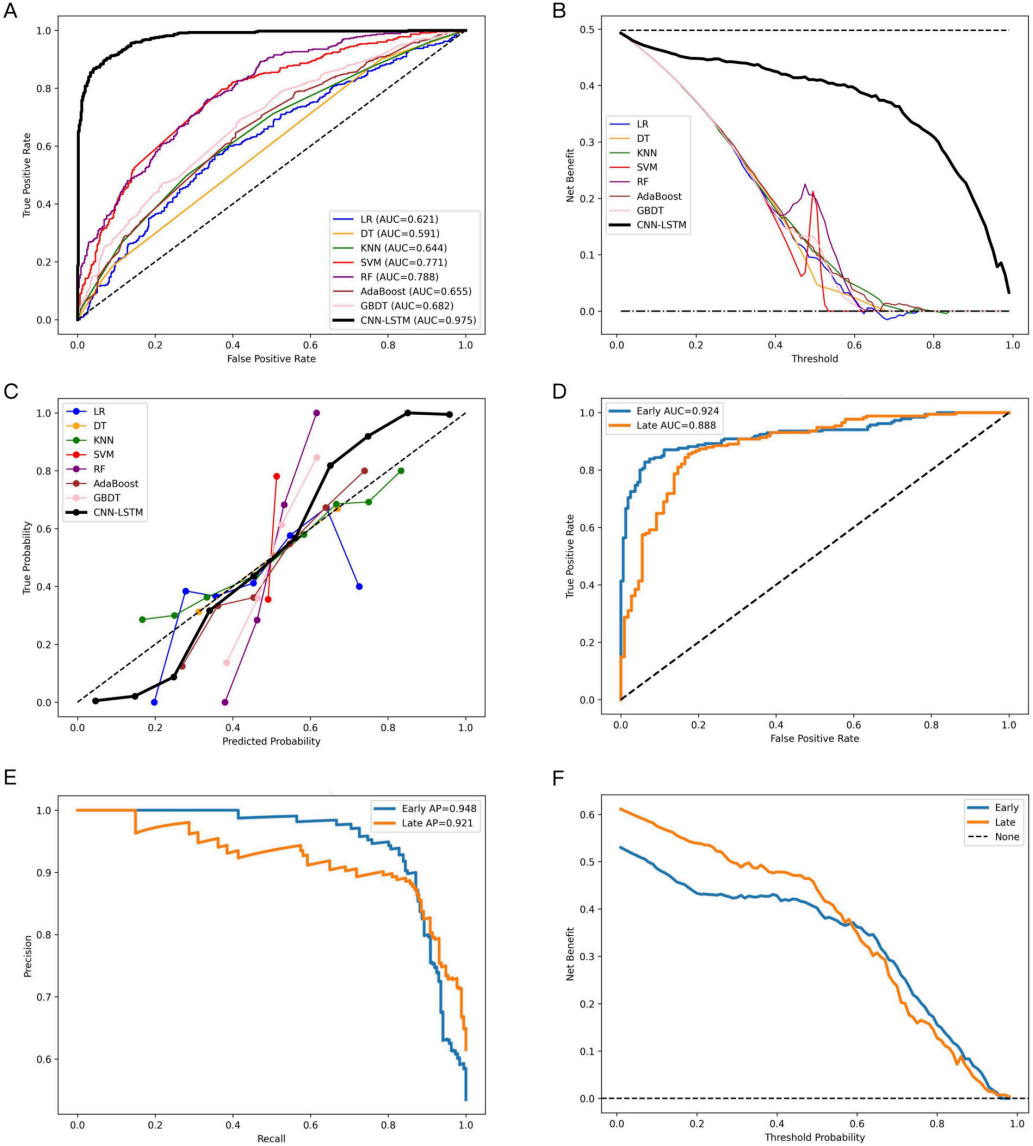
Panel **(A)** shows a line graph comparing the ROC curves of eight machine learning models, each with different AUC values, for true positive rate versus false positive rate. Panel **(B)** displays a line graph of net benefit versus threshold for the same models. Panel **(C)** presents a calibration plot comparing true probability to predicted probability for all models. Panel **(D)** illustrates ROC curves for early and late models, with respective AUC values. Panel **(E)** features a precision-recall curve for early and late models, noting their AP scores. Panel **(F)** compares net benefit as a function of threshold probability for early and late models.

We compared the proposed CNN-LSTM model with seven typical machine learning models, including logistic regression (LR), decision tree (DT), k-nearest neighbor (KNN), support vector machine (SVM), random forest (RF), AdaBoost, and gradient boosting decision tree (GBDT). Performance metrics including accuracy, F1-score, and AUC with 95% confidence intervals (CI) were evaluated across training, validating, and testing sets (Table 2). Overall, the proposed CNN-LSTM model outperformed all comparison models across training, validating, and testing datasets. On the testing set, CNN-LSTM achieved the best overall performance with accuracy of 0.970 (95%CI: 0.961–0.977), F1-score of 0.970 (95%CI: 0.961–0.977), and AUC of 0.975 (95%CI: 0.962–0.988). In addition, RF and SVM also showed relatively better performance among the traditional ML approaches. In contrast, LR, DT, KNN, AdaBoost, and GBDT yielded relatively lower predictive ability with most AUC values below 0.70 on the testing set. The SVM model achieved a moderate performance with testing AUC of 0.771 (95%CI: 0.725–0.826), and RF achieved testing AUC of 0.788 (95%CI: 0.728–0.844), both of which were still significantly lower than that of the proposed CNN-LSTM model (Table 3, Figure 4A).

**Table 3:** Performance comparison of the proposed CNN-LSTM model with other classifiers.

Classifier	Datasets	Accuracy (95%CI)	F1 (95%CI)	AUC (95%CI)
CNN-LSTM	Training	0.970 (0.960–0.979)	0.970 (0.961–0.978)	0.975 (0.962–0.988)
	Validating	0.970 (0.962–0.977)	0.970 (0.960–0.976)	0.970 (0.961–0.978)
	Testing	0.970 (0.961–0.977)	0.970 (0.960–0.977)	0.975 (0.962–0.988)
LR	Training	0.655 (0.624–0.681)	0.655 (0.623–0.679)	0.656 (0.625–0.678)
	Validating	0.652 (0.621–0.674)	0.653 (0.622–0.675)	0.650 (0.623–0.671)
	Testing	0.621 (0.590–0.648)	0.621 (0.591–0.647)	0.621 (0.593–0.646)
DT	Training	0.600 (0.572–0.628)	0.697 (0.669–0.725)	0.668 (0.640–0.692)
	Validating	0.600 (0.571–0.627)	0.650 (0.595–0.695)	0.600 (0.570–0.628)
	Testing	0.591 (0.562–0.617)	0.611 (0.582–0.638)	0.591 (0.563–0.616)
KNN	Training	0.647 (0.609–0.676)	0.648 (0.602–0.686)	0.708 (0.673–0.744)
	Validating	0.600 (0.570–0.629)	0.600 (0.571–0.628)	0.600 (0.572–0.627)
	Testing	0.644 (0.618–0.669)	0.644 (0.619–0.668)	0.644 (0.617–0.667)
SVM	Training	0.880 (0.856–0.902)	0.880 (0.855–0.903)	0.880 (0.857–0.901)
	Validating	0.855 (0.828–0.877)	0.857 (0.834–0.892)	0.855 (0.829–0.878)
	Testing	0.745 (0.712–0.774)	0.745 (0.713–0.773)	0.771 (0.725–0.826)
RF	Training	0.897 (0.878–0.914)	0.893 (0.876–0.915)	0.867 (0.842–0.891)
	Validating	0.700 (0.671–0.726)	0.700 (0.672–0.725)	0.700 (0.673–0.728)
	Testing	0.788 (0.750–0.833)	0.788 (0.737–0.872)	0.788 (0.728–0.844)
AdaBoost	Training	0.636 (0.600–0.668)	0.647 (0.604–0.682)	0.670 (0.626–0.707)
	Validating	0.662 (0.633–0.688)	0.662 (0.647–0.688)	0.664 (0.625–0.691)
	Testing	0.658 (0.630–0.682)	0.665 (0.632–0.691)	0.655 (0.621–0.683)
GBDT	Training	0.798 (0.774–0.822)	0.797 (0.773–0.824)	0.884 (0.861–0.901)
	Validating	0.731 (0.704–0.756)	0.700 (0.671–0.726)	0.730 (0.702–0.755)
	Testing	0.611 (0.582–0.638)	0.608 (0.579–0.635)	0.682 (0.651–0.710)

The clinical utility of the CNN-LSTM model, as assessed through decision curve analysis (DCA, Figure 4B), revealed its superior practical value in clinical decision-making, offering the highest net benefit across a broad range of threshold probabilities when compared to alternative models. This underscores its exceptional potential for enhancing clinical decision-making in real-world settings. Additionally, calibration analysis (Figure 4C) demonstrated excellent alignment between the model's predicted probabilities and observed outcomes, highlighting its strong reliability and calibration performance.

The predictive efficacy of the CNN-LSTM model was further validated by evaluating its performance across early-stage (Stage II) and late-stage (Stage III + IV) patient cohorts, as shown in Figure 4D. Subgroup-specific analysis revealed that the model performed robustly in both early-stage and late-stage cohorts. In the early-stage cohort, the model achieved an area under the receiver operating characteristic curve (AUC) of 0.924, while in the late-stage cohort, it reached 0.888. Both values were not only high but also clinically significant (Figure 4D). Furthermore, precision-recall curves (Figure 4E) confirmed the model's powerful discriminative ability, with area under the precision-recall curve (AP) values of 0.948 and 0.921 for the early-stage and late-stage cohorts, respectively.

Decision curve analysis (DCA) was performed to evaluate the clinical utility of the CNN-LSTM model in both early-stage and late-stage cancer cohorts (Figure 4F). Across most clinically relevant threshold probabilities (0.1–0.9), the net benefit of the model remained consistently positive and superior to the “treat-none” strategy. Notably, the late-stage subgroup exhibited higher net benefit at lower thresholds, while the early-stage subgroup maintained stable clinical utility across a broader range of thresholds.

These findings confirm that the CNN-LSTM model not only outperforms conventional machine learning models in overall predictive performance but also demonstrates strong generalizability and consistent clinical utility for guiding risk stratification and cardioprotective interventions across both early-stage and late-stage cancer subgroups.

### 3.5 Analysis of predictive factors

**Figure 5:**
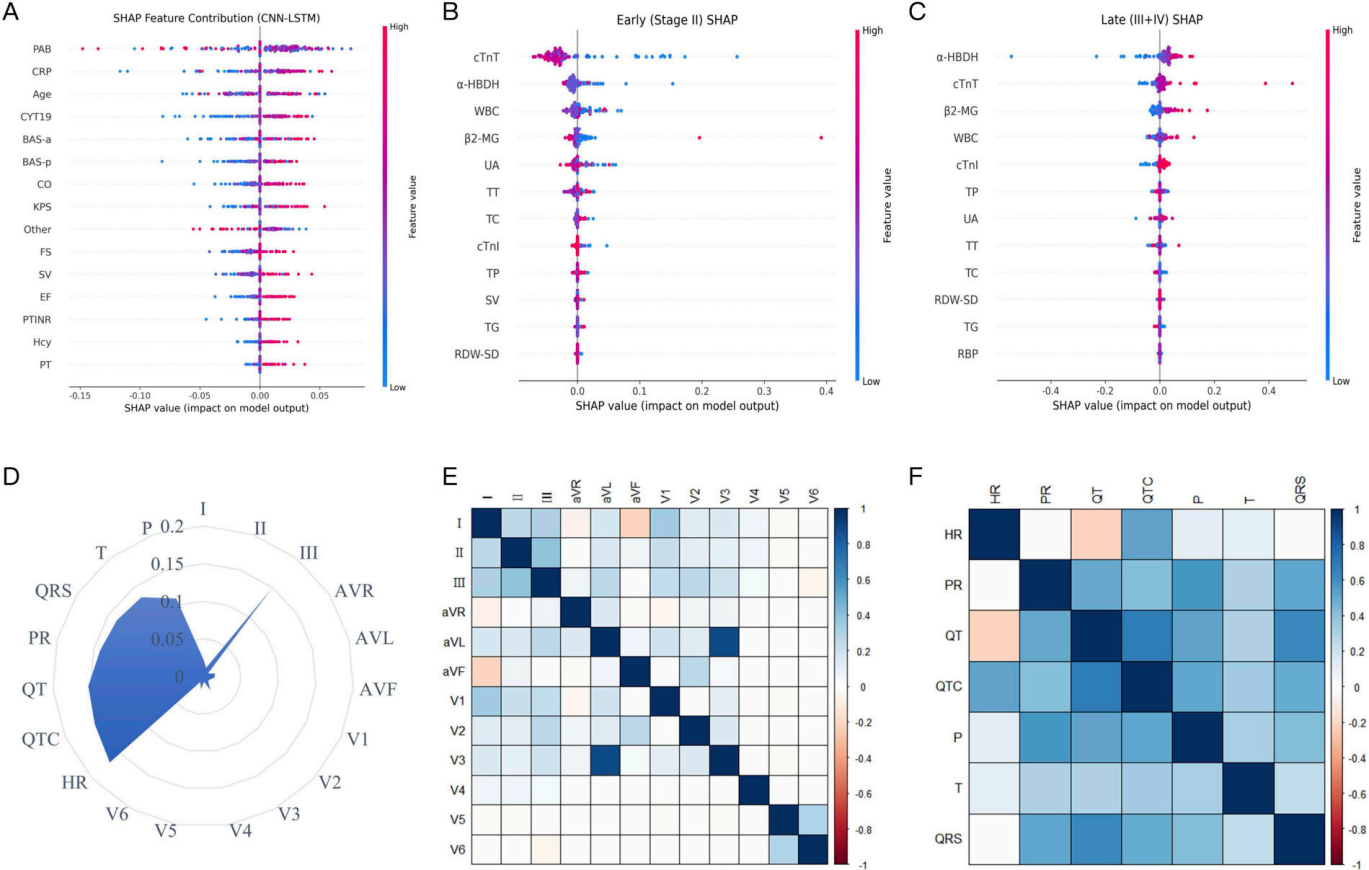
Panel **(A)** presents a dot plot illustrating SHAP feature contributions for a CNN-LSTM model, with variables such as PAB, CRP, Age, and others, and coloring indicating feature value. Panel **(B)** displays a similar SHAP value plot for early disease stage, focusing on laboratory features like cTnT and α-HBDH. Panel **(C)** shows SHAP values for late disease stage, with a changed feature importance order. Panel **(D)** is a radar chart mapping different ECG features. Panel **(E)** is a correlation heat map among multiple ECG lead variables, with intensity reflecting the strength and direction of correlation. Panel **(F)** is a correlation heat map among several ECG measurement parameters.

To enhance model interpretability and identify key predictive features, SHapley Additive exPlanations (SHAP) and correlation-based analyses were applied to the proposed CNN–LSTM framework. The results are presented in Figure 5. As shown in Figure 5A, the global SHAP summary plot illustrates the overall contribution of clinical and physiological features to model predictions in the CNN–LSTM model. Each point represents the impact of a feature on the model output across all samples. The most influential features included platelet albumin binding protein (PAB), C-reactive protein (CRP), age, cytokeratin 19 (CYFRA21-1, CYT19), and baseline albumin-related markers, followed by clinical status indicators such as Karnofsky Performance Status (KPS), cardiac output (CO), and heart failure-related variables (e.g., PT, Hcy).

As shown in Figure 5B, SHAP analysis stratified by early-stage (Stage II) cancer patients reveals that cardiac troponin T (cTnT), *α*-hydroxybutyrate dehydrogenase (*α*-HBDH), white blood cell count (WBC), and *β*2-microglobulin (*β*2-MG) are the most influential predictors in early disease stages. In addition, uric acid (UA), total protein (TP), triglycerides (TG), and red cell distribution width (RDW-SD) also contribute to risk stratification, indicating that inflammatory and metabolic dysregulation play important roles in early Ca-CHD development. In contrast, Figure 5C shows SHAP feature importance in advanced-stage (Stage III–IV) patients. The dominant features include *α*-HBDH, cTnT, *β*2-MG, WBC, and cardiac troponin I (cTnI), followed by coagulation and metabolic markers such as prothrombin time (PT), uric acid (UA), triglycerides (TG), and total cholesterol (TC).

As shown in Figure 5D, radar plot analysis of ECG-derived features demonstrates that heart rate (HR), Q-T interval (QT), corrected QT interval (QTc), and QRS complex contribute most significantly to model predictions: the weights of top leads are: I (0.023), V3 (0.0204), V5 (0.0166), heart rate (HR, 0.168), Q-T interval (QT, 0.153) and QTC period (0.157). Among them, HR shows the highest contribution, followed by QT and QTc, indicating that cardiac rhythm and repolarization abnormalities are key electrophysiological signals associated with Ca-CHD risk. Figure 5E presents the correlation matrix of 12-lead ECG signals. Strong inter-lead correlations were observed, particularly between anatomically adjacent leads such as aVL and V3, as well as V5 and V6. These correlations reflect spatial coherence of cardiac electrical activity and support redundancy-aware feature learning in the CNN module. As shown in Figure 5F, significant correlations were observed among ECG interval features. The QT interval showed strong correlations with QRS duration, while PR interval was moderately correlated with P-wave duration. Additionally, QTc demonstrated consistent associations with both QT and HR, indicating that ventricular repolarization dynamics are closely linked to cardiac rhythm abnormalities in Ca-CHD patients. These findings suggest that cardiac injury markers and coagulation dysfunction become more prominent in advanced disease stages.

## 4 Discussion

In this study, we developed a hybrid CNN-LSTM model that fuses multi-modal data from electronic health records (EHRs) and 12-lead electrocardiogram (ECG) for predicting new-onset coronary heart disease in cancer patients (Ca-CHD). The proposed model achieved an AUC of 0.975 (95%CI: 0.962–0.988) on the test set, outperforming seven conventional machine learning models including logistic regression, decision tree, KNN, SVM, random forest, AdaBoost, and GBDT. Subgroup analyses confirmed robust performance in both early-stage (Stage II, AUC = 0.924) and late-stage (Stage III + IV, AUC = 0.888) cohorts, with favorable calibration and clinical net benefit across threshold probabilities. These findings support the value of multi-modal deep learning for cardio-oncology risk stratification.

Our results align with emerging evidence that cardiovascular disease is a leading complication and non-oncologic cause of death in cancer populations. Patients with malignancies share common pathogenic mechanisms with coronary heart disease, including chronic inflammation, endothelial dysfunction, hypercoagulability, and metabolic disorders, all of which can be exacerbated by chemotherapy, targeted therapy, and radiotherapy. Clinical risk factors identified in our baseline analyses—advanced age, elevated cTnI, prolonged prothrombin time (PT), increased LDL, and reduced stroke volume (SV), consistent with established markers of subclinical myocardial injury and thrombotic risk in cancer patients (12, 32). Such disruptions create a pathophysiological substrate for accelerated coronary artery disease and acute cardiovascular events during anticancer treatment.

The superior performance of the CNN-LSTM model highlights the advantage of integrating heterogeneous clinical information. Unlike traditional models that rely on limited structured variables, our framework jointly learns from textual clinical notes, laboratory biomarkers, and 12-lead ECG signals. The CNN component effectively extracts spatial patterns from ECG waveforms and multi-dimensional bio signals, while the LSTM module captures temporal dependencies in sequential physiological changes. This hybrid design enables more comprehensive feature representation than single-modality approaches, consistent with recent studies demonstrating that multi-modal fusion improves cardiovascular risk prediction in oncology populations (24–26).

SHAP-based interpretability revealed the key drivers of model prediction. Prothrombin time (PT), one of the sensitivity indexes of thrombosis, reflects prothrombotic status, which increases venous and arterial thromboembolic risk in malignancy (33). Cardiac patients with tumor may increase the risk for the formation of arterial or venous thrombosis. The characteristics of ECG signals have association with cancer progression since tumor patients mainly present with sympathetic imbalance, ventricular recovery time increases inconsistently (34, 35). HR and QTC are important as well. Increases in cTnI and NPs were detected along with prolongation of the QTc interval in the cancer situation (12). The results suggest that higher performance of Rv1 and Sv5 leads of ECG are closely related with the disease. Lead I reflect changes in the anterior wall of the left ventricle, while chest lead V5 reflects changes in the left ventricle. V5 lead has the highest sensitivity to detect myocardial ischemia, ventricular hypertrophy, and other diseases (36). Optimizing the number of leads by choosing the most significant ones helps the network to achieve the best performance. Collectively, SHAP-based interpretation and ECG correlation analyses provide complementary evidence for model interpretability. These results demonstrate that both biochemical biomarkers (e.g., PAB, PT, CRP) and electrophysiological features (e.g., QT, QTc, HR) contribute synergistically to the prediction of cancer-associated CHD, thereby enhancing the clinical interpretability and robustness of the proposed multimodal CNN–LSTM framework.

Notably, coagulation-related markers exhibited prominent feature importance in our model, supporting a critical pathogenic cascade involving cancer-induced hypercoagulability, subsequent thrombosis formation, and eventual new-onset coronary heart disease in cancer patients. Malignancy itself triggers a systemic prothrombotic state via tumor-derived procoagulants, inflammatory cytokines, and endothelial injury, which greatly elevates the risk of arterial thrombosis and subsequent acute coronary events (32). In our SHAP analysis, prothrombin time (PT) and fibrin degradation products (FDP) were identified as key predictors, consistent with their roles in reflecting impaired coagulation balance and ongoing fibrinolytic activity in cancer. Prolonged PT indicates impaired hepatic synthesis or consumption of clotting factors, while elevated FDP reflects recent or ongoing thrombotic processes (37). Together, these markers highlight that thrombosis and coagulation dysfunction are core intermediate steps linking cancer progression to the development of *de novo* CHD. In addition to coagulation indicators, cardiac injury markers (cTnI), lipid disorders (LDL), and inflammatory markers (CRP) also contributed strongly to predictions, suggesting that myocardial damage, metabolic disturbance, and chronic inflammation act alongside hypercoagulability to promote coronary artery disease in cancer.Stage-stratified analyses further revealed distinct predictive profiles. Early-stage cancer was more strongly associated with inflammatory and metabolic markers such as cTnT, *α*-HBDH, and *β*2-MG, whereas late-stage disease was dominated by markers of myocardial injury, coagulopathy, and ECG repolarization abnormalities. This stage-dependent shift suggests that cardiovascular risk mechanisms evolve with cancer progression, justifying personalized risk assessment strategies (38).

Our model's stable performance across early and late subgroups demonstrates its potential for real-world clinical implementation across disease stages. From a clinical perspective, the proposed multi-modal CNN-LSTM model provides a transparent and actionable tool for proactive Ca-CHD screening. By incorporating routinely collected EHR and ECG data, the system can be integrated into existing oncology workflows without additional invasive testing. Decision curve analysis confirmed that the model improves net clinical benefit compared with a “treat-all” strategy across most clinically relevant thresholds, supporting its utility for shared decision-making and targeted cardio protection.

Several limitations should be acknowledged. First, this is a single-center retrospective study, which may limit generalizability. Second, the sample size, while sufficient for model development, would benefit from external validation in multi-center cohorts. Third, thromboelastographic (TEG) data were not available in this study. As a comprehensive tool for evaluating global coagulation function, TEG can reflect platelet function, clot formation kinetics, and fibrinolytic activity in real time. The lack of TEG limited our ability to fully characterize the hypercoagulable state and its direct contribution to thrombosis and CHD. This represents an important limitation that should be addressed in future prospective studies by incorporating TEG and other advanced coagulation monitoring modalities. Future studies should prospectively validate the model, explore real-time edge deployment for wearable monitoring, and investigate whether model-guided intervention improves cardiovascular outcomes in cancer patients.

## 5 Conclusion

In conclusion, the multimodal CNN-LSTM model achieves good and stable predictive performance for new-onset coronary heart disease in cancer patients, with an overall AUC of 0.975 (95%CI: 0.962–0.988), and maintains favorable discriminative ability in both early-stage (AUC = 0.924) and late-stage (AUC = 0.888) subgroups. The key predictive factors include age, PAB, CRP, cTnI, PT, as well as heart rate (HR), QT interval and QTc interval. This model provides a reliable and interpretable tool for early risk stratification, and the identified biomarkers involving coagulation, myocardial injury, inflammation and electrophysiological abnormalities offer important mechanistic insights for the development of personalized cardioprotective strategies in cancer patients.

## Data Availability

The raw data supporting the conclusions of this article will be made available by the authors, without undue reservation.
